# The Surgical Treatment of Severe Dysenteric Enterocolitis

**Published:** 1911-01

**Authors:** M. E Poucel

**Affiliations:** Marseilles, France


### The Surgical Treatment of Severe Dysenteric Enterocolitis.

By M. E POUCEL, Marseilles, France.

(*La Tribune Medicale* American Edition)

THE dangers of severe cases of dysenteric enterocolitis are well known. These cases are very frequently fatal, whether they be of the hemorrhagic, ulcerating, perforating or gangrenous type. The mortality varies from 20 to 50 per cent.

according to the climate, the locality and to the pathogenic agent which is the cause of the infection. Of these a specially virulent type of the ameba coli and a form of the streptococcus pyogenes are the most prominent. It is well known that medical treatment is of but little use in these cases, although they may be excellent in the milder forms.

Intestinal antiseptics, Dover's powder, enemas of silver nitrate solution are useless when the diarrhea has become extreme in severity and when there is actual ulceration and loss of tissue in the intestine with fever and sepsis.

For a long time I have been of the opinion that the only treatment in such cases is to establish a large artificial anus and I performed this operation twelve years ago in a very severe case. I made a mistake, however, in the fact that I made a temporary anus. The trouble is that no matter how large such an opening may be, some of the contents insists on following the normal route and thus prevents cure.

So, after a period of improvement which lasted two weeks, the severe symptoms recurred in my patient, who preferred to die of hunger rather than to suffer the agonies which were aggravated by the taking of food. The patient died in about a month, and I am convinced she could have been saved by a permanent anus. Accordingly, I made it my business to apply this rule at the very next opportunity. On February 3d I was called to see a patient presenting an ideal case for the application of this method. He had a severe type of dysenteric enterocolitis, from which he was dying. There was a temperature of 39.5° C., a constant tenesmus, 30 or 40 stools a day, containing blood, and all the other symptoms of the extreme form of this condition. The patient had been condemned to death by a famous clinician who thought that he might have a cancer.

There was no question of cancer in this case, but of a dysenteric condition which was apt to be fatal. On the following day I made an artificial anus with two orifices, one in the ileum, the other in the cecum. The appendix, which was long and thick but not inflamed, was removed.

On the next day the large intestine was washed with a solution of iodine containing one quarter of 1 per cent. of the drug. The tenesmus, the blood clots and the fever disappeared on the same day. On the 7th and 8th the patient's appetite returned and on the 10th he got up in bed. The lesion in the rectum had completely disappeared.

This treatment can be applied to all the severe forms of dysentery, no matter what their cause, even when they are due to tropical parasites. In the latter cases the affection involves a larger surface, and includes the ileum, and so the treatment is not so prompt in its effects. Yet the exclusion of the colon, which is the principal seat of the trouble, contributes a great deal to the improvement of the condition.

This operation removes the chief focus of infection and allows the ulcerations to heal, provided, of course, that they are not tuberculous or cancerous. Furthermore, the operation arrests the hemorrhage and renders antisepsis of the ileum possible. It may not be considered out of place to say that the surgical treatment in question may be of value in all forms of reinfection in which the intestine is the primary focus, as for example in typhoid fever. It is worth while to experiment with this method, at least in desperate cases. Of course, a single case is not sufficient to formulate conclusions, yet the method seems perfectly rational.

You will note that it is not an ordinary case of artificial anus of which I speak, but that I am presenting to you an idea of the method of treatment which I think may save a great many lives which are lost every day in all climates as the result of severe cases of dysentery.

The operation is so simple that it can be performed on an almost moribund patient under cocaine. If the patient after convalescence finds it tedious to wear the necessary appliance, he can be quite easily relieved of his infirmity.

